# Naphthalene-Containing Epoxy Resin: Phase Structure, Rheology, and Thermophysical Properties

**DOI:** 10.3390/polym16233264

**Published:** 2024-11-24

**Authors:** Svetlana O. Ilyina, Irina Y. Gorbunova, Anastasiya Y. Yadykova, Anna V. Vlasova, Michael L. Kerber, Sergey O. Ilyin

**Affiliations:** 1 A.V. Topchiev Institute of Petrochemical Synthesis, Russian Academy of Sciences, 29 Leninsky Prospect, 119991 Moscow, Russia; 2 Department of Plastics Processing Technology, D. Mendeleev University of Chemical Technology of Russia, 9 Miusskaya Square, 125047 Moscow, Russia

**Keywords:** epoxy resin, naphthalene, diaminodiphenyl sulfone, fungicides, phase-change materials, miscibility, phase separation, rheology, epoxy curing, calorimetry

## Abstract

Naphthalene is a fungicide that can also be a phase-change agent owing to its high crystallization enthalpy at about 80 °C. The relatively rapid evaporation of naphthalene as a fungicide and its shape instability after melting are problems solved in this work by its placement into a cured epoxy matrix. The work’s research materials included diglycidyl ether of bisphenol A as an epoxy resin, 4,4′-diaminodiphenyl sulfone as its hardener, and naphthalene as a phase-change agent or a fungicide. Their miscibility was investigated by laser interferometry, the rheological properties of their blends before and during the curing by rotational rheometry, the thermophysical features of the curing process and the resulting phase-change materials by differential scanning calorimetry, and the blends’ morphologies by transmission optical and scanning electron microscopies. Naphthalene and epoxy resin were miscible when heated above 80 °C. This fact allowed obtaining highly concentrated mixtures containing up to 60% naphthalene by high-temperature homogeneous curing with 4,4′-diaminodiphenyl sulfone. The initial solubility of naphthalene was only 19% in uncured epoxy resin but increased strongly upon heating, reducing the viscosity of the reaction mixture, delaying its gelation, and slowing cross-linking. At 20–40% mass fraction of naphthalene, it almost entirely retained its dissolved state after cross-linking as a metastable solution, causing plasticization of the cured epoxy polymer and lowering its glass transition temperature. At 60% naphthalene, about half dissolved within the cured polymer, while the other half formed coarse particles capable of crystallization and thermal energy storage. In summary, the resulting phase-change material stored 42.6 J/g of thermal energy within 62–90 °C and had a glass transition temperature of 46.4 °C at a maximum naphthalene mass fraction of 60% within the epoxy matrix.

## 1. Introduction

Naphthalene is an aromatic hydrocarbon consisting of two fused benzene rings. In appearance, it is a white crystalline substance with a characteristic pungent odor. There are many sources of naphthalene in the environment. Primarily, it is a crude oil component and product of natural combustion [1,2]. Because of its low but notable volatility, naphthalene-containing mothballs have long been used to protect various objects and materials from insects and rodents such as moths, ants, cockroaches, and mice. It effectively kills and repels these pests, preventing their emergence, and is applicable for keeping and protecting household items, fabrics, and museum exhibits [3,4,5,6]. In addition, naphthalene can be helpful as a fungicide in controlling blood-sucking insects [7], pests, and plant pathogens [8,9].

Nevertheless, naphthalene is toxic to humans and the environment. When entering the soil and water, naphthalene leads to contamination, threatening the lives of various species of animals and plants [10]. For humans, exposure to naphthalene may lead to poisoning and different diseases. Inhalation of vapors of this substance causes headaches, lacrimation, coughing, nausea, vomiting, diarrhea, and even seizures. Prolonged exposure to naphthalene may damage or destroy erythrocytes and trigger cancer development [11,12,13,14,15,16]. Meanwhile, naphthalene is often used in the pure form of flakes, mothballs, or tablets. As a result, its vapors are produced in high concentrations because of relatively rapid evaporation, which can harm human and pet health.

A solution to the relatively high evaporation rate of naphthalene may be to place it in a polymer shell or matrix, which would increase the lifetime of the fungicide and reduce its toxic effects. In this case, a less labor-intensive method is to obtain a polymer matrix with dissolved naphthalene rather than capsules with a polymer shell, whose production is technologically complex and costly [17,18,19]. When selecting a polymer matrix, thermosets are preferable to thermoplastics because of lower viscosity. A low-viscosity thermosetting resin (e.g., epoxy) may allow for better dispersion or dissolution of a crystallizable substance in a solid or molten state [20,21]. Moreover, commercially produced epoxy resin contains aromatic fragments of bisphenol A, which may contribute to the dissolution of naphthalene at elevated temperatures. After that, the naphthalene-containing epoxy resin can be shaped to any desired form and then cured. Building materials, furniture elements, and storage containers obtained this way will have fungicidal properties to repel moths and other insects while being safe because of the low release rate of naphthalene into the environment.

In addition, a peculiarity of naphthalene is a relatively low melting point with a rather high thermal effect of about 142–153 J/g at 80 °C. If naphthalene crystallizes in the cured epoxy medium, the resulting composite could be a phase-change material (PCM) to store thermal energy and improve indoor heating efficiency [22,23,24,25,26]. Most PCMs absorb and then release heat in transition between liquid and solid states, thus using latent thermal energy [27,28]. However, typical unmodified PCMs are not shape-stable due to the transformation into flowable liquid upon melting [29], attracting significant attention to various gelators [30,31,32], nanoparticles [33,34,35], and polymers [36,37] introduced in their composition to provide a solid-like behavior. In this respect, cross-linking of naphthalene-containing epoxy resin guarantees its shape stability [38], potentially yielding PCM building elements [39] for storing heat during the warm hours of the day and then releasing it at night [40,41,42,43], for example, in epoxy tiles or floors.

Previously, multifunctional epoxy resins containing naphthalene moieties in their structure have been synthesized for electronics, including the creation of microchips and semiconductors [44,45,46,47,48,49,50,51,52,53]. The obtained naphthyl-containing epoxy resins have higher glass transition temperature, thermostability, and storage modulus, acquiring lower thermal expansion coefficient and moisture absorption. In addition, the naphthalene moiety can act as a mesogen, allowing the synthesis of a liquid crystalline epoxy resin for use in construction adhesives and matrices [54,55]. Moreover, epoxy derivatives of naphthalene have been synthesized, which have luminescent properties and are helpful as luminescent materials, light-emitting diodes, and organic semiconductors [56]. Thus, all the works on epoxy resin and naphthalene have focused on the chemical incorporation of naphthalene as a naphthyl moiety into the epoxy resin composition. Straight mixtures of naphthalene and epoxy resin, their phase state, and physicochemical properties have not been previously studied.

This work aims to obtain epoxy compositions containing naphthalene as a component in a wide concentration range to evaluate their phase state, morphology, and physicochemical properties critical for shaping products and their subsequent applications. The novelty of the research consists of obtaining epoxy compositions containing pure naphthalene for the first time by controlled phase separation initiated by curing and studying the miscibility of components, the phase state of the resulting blends, their morphology, and their thermophysical and rheological properties before, during, and after the curing.

## 2. Materials and Methods

### 2.1. Materials

Diglycidyl ether of bisphenol A (DER-330, Dow Chemical, Midland, MI, USA) containing 180.5 g/mol-eq of epoxy groups served as a thermosetting resin. The curing agent was 4,4′-diaminodiphenyl sulfone (Sigma-Aldrich, Steinheim, Germany), containing 62.1 g/mol-eq of amine groups. The resin/hardener ratio was stoichiometric: 74.4/25.6 wt%/wt% [57]. Naphthalene with a melting point of 80 °C and purity of 99% (Sigma-Aldrich) was used as a fungicidal and phase-change agent. The naphthalene mass fraction in the resin/hardener blend was 0, 20, 40, or 60 wt%. The mixture containing 80% naphthalene was not investigated, as its curing resulted in a dispersion of the cured epoxy polymer in the naphthalene matrix.

All blends were prepared by mixing the components on a magnetic stirrer at 100 °C for 30 min. Then, a part of their mass was cooled to 25 °C for rheological and calorimetric tests, while the other part was poured into silicone molds for subsequent curing. The silicone molds containing the blends were placed in a sealed glass container to prevent the vaporization of naphthalene and then put in an oven for 3 h at 180 °C.

### 2.2. Methods

The miscibility of naphthalene and epoxy resin was evaluated by laser interferometry, described in detail elsewhere [58,59]. The naphthalene/resin diffusion cell was stepwise heated from 25 °C to 85 °C and then slowly cooled to 25 °C. A 3.5× magnification objective lens and a digital camera with a 12-megapixel 1/1.7-inch IMX226 CMOS image sensor (Sony, Tokyo, Japan) were used to capture naphthalene/resin interferograms. A diode laser KLMA532-15-5 (FTI-Optronic, St. Petersburg, Russia) was a light source and had a wavelength of 532 nm.

Rheological properties were studied on a DHR-2 rotational rheometer (TA Instruments, New Castle, DE, USA). Uncured samples were investigated at 25 °C using a cone/plate measuring unit with an angle between the cone and plate of 2° and a plate diameter of 40 mm. Their flow curves were obtained by a stepwise increase in shear rate from 10^−4^ to 1000 s^−1^ with a measurement time for each shear rate of at least 20 s. Linear viscoelasticity was investigated at a strain amplitude of 0.1% and an angular frequency range of 0.0628–628 rad/s. A plate/plate measuring unit with 25 mm plate diameters was used to study the temperature dependence of samples’ effective viscosity at a heating rate of 2 °C/min, a temperature range of 25–250 °C, and a constant shear rate of 10 s^−1^. Rheological characteristics were calculated according to the known equations [60,61]. The relative error in the measurement was not more than 5%.

Differential scanning calorimetry (DSC) was carried out in an argon medium on an MDSC 2920 calorimeter (TA Instruments). The heat flow of uncured samples was measured when heating up to 280 °C at a rate of 2 °C/min to determine the thermal effect of epoxy cross-linking and the naphthalene’s influence on its rate. In addition, pre-cured samples were examined at a temperature increasing from 20 °C to 210 °C at a rate of 10 °C/min to find the glass transition temperature of the epoxy matrix and melting point of dispersed naphthalene. The accuracy in determining transition temperatures was ±0.2 °C, and the relative error in their enthalpies did not exceed 5%.

Microphotographs of samples cured between two cover glasses were captured using a 10× magnification lens on a digital camera with a Sony IMX226 image sensor. Scanning electron microscopy (SEM) of cross-sectional areas of cured samples after crashing was performed on a Phenom XL G2 microscope (Thermo Fisher Scientific, Eindhoven, The Netherlands) under an accelerating voltage of 15 kV and pressure of 60 Pa. A 5 nm layer of gold was pre-applied on top of investigated surfaces by ion-plasma sputtering on a 108 Auto device (Cressington Scientific Instruments, Watford, UK).

## 3. Results and Discussion

### 3.1. Phase State of Naphthalene/Epoxy Resin Blends

Naphthalene may dissolve, partially dissolve, or remain as crystals when mixed with epoxy resin, and the phase state of the resultant mixture may be temperature-dependent. In turn, the phase state of the naphthalene-containing blend (a solution, an emulsion, or a suspension) will determine its rheological properties and ability to be shaped into final products. Therefore, it is necessary to evaluate the temperature dependence of naphthalene solubility in epoxy resin, which can be performed by laser interferometry when two substances are brought into contact in a thin layer to study their mutual diffusion [62].

**Figure 1:**
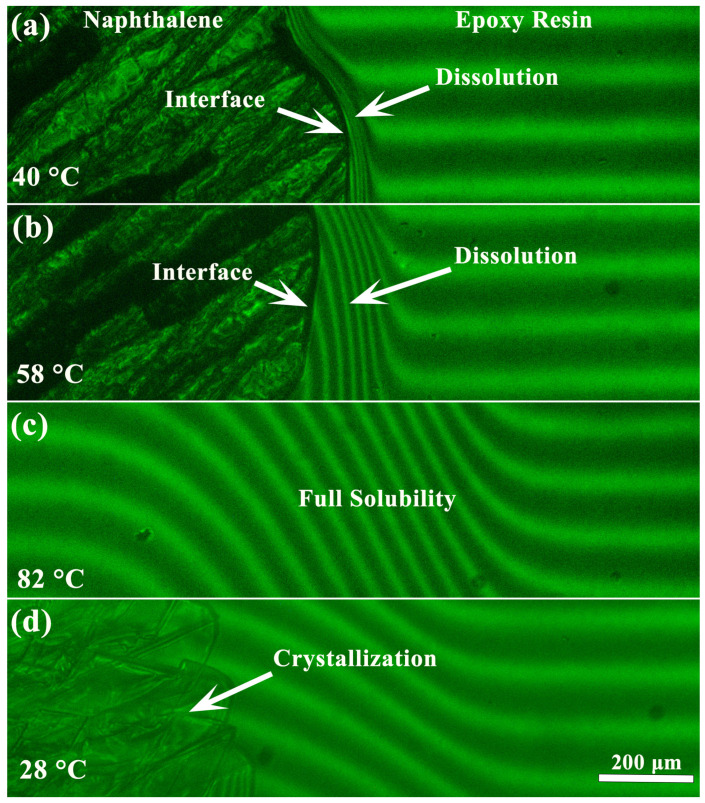
Interferograms of a mutual diffusion zone of naphthalene (**left**) and epoxy resin (**right**) during sequential heating to 40 (**a**), 58 (**b**), and 82 °C (**c**) and then cooling to 28 °C (**d**).

At low temperatures, naphthalene is solid, and its crystals are visible in interferograms (Figure 1, left). Heating of the naphthalene/epoxy resin system to 40 °C leads to intense bends of interference fringes along the interfacial boundary on the epoxy resin’s side (Figure 1a), indicating the dissolution of naphthalene in the resin. With increasing temperature, the solubility of naphthalene increases, causing a broadening of the mutual diffusion zone (a zone where interference fringes bend) and a shift of the phase boundary to the naphthalene’s side due to its dissolution (Figure 1b). An increase in the temperature above 80 °C causes naphthalene to melt, and the diffusion zone becomes continuous, implying complete mutual solubility of naphthalene and epoxy resin (Figure 1c). Subsequent cooling induces crystal formation but not in the whole diffusion zone (Figure 1d), indicating a partial solubility of naphthalene in the epoxy resin even at low temperatures. Thus, the phase state of the epoxy resin/naphthalene system is characterized by a solid–liquid (crystalline) equilibrium [63].

**Figure 2:**
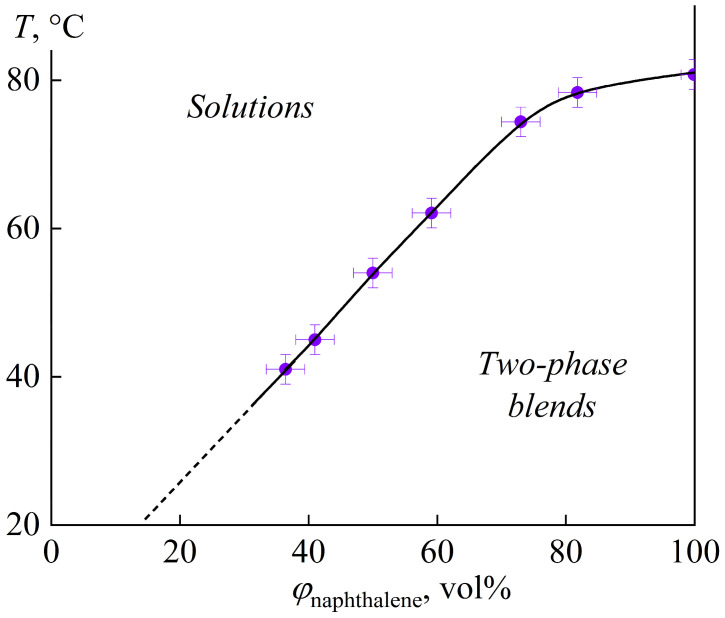
Phase diagram for mixtures of epoxy resin and naphthalene.

Based on the set of interferograms at different temperatures, a phase diagram for epoxy resin and naphthalene can be plotted (Figure 2). The phase diagram reflects the depression of the crystallization temperature of naphthalene. A solution region exists in an area of high temperatures and low naphthalene contents (*w*_naph_), whereas low temperatures and high *w*_naph_ lead to two-phase systems consisting of naphthalene crystals in its saturated solution in epoxy resin. The interpolation of the liquidus line down to 25 °C gives a solubility of naphthalene in epoxy resin equal to 19 vol% at this temperature, corresponding to 19 wt% due to the close densities of these two substances.

Thus, naphthalene dissolves completely in epoxy resin only at elevated temperatures. This fact means that a high-temperature curing is necessary to obtain miscible blends. However, an ordinary commercial hardener (e.g., an aliphatic amine) will lead to almost instantaneous cross-linking at elevated temperatures with high internal stresses and cracking of the cured blend. A hardener that cross-links the epoxy resin only at elevated temperatures is required. In addition, naphthalene dissolved in the cured resin may significantly reduce its heat deflection temperature, indicating the need for a hardener that increases the glass transition temperature of the cross-linked epoxy polymer. 4,4′-Diaminodiphenyl sulfone meets both criteria, curing an epoxy resin at 150–180 °C [64] and giving a product with the highest glass transition temperature of about 180–200 °C [65,66]. Nevertheless, only low-temperature mixing prevents premature curing, implying the significance of the rheological behavior of epoxy/hardener/naphthalene mixtures under these conditions.

### 3.2. Rheological Properties of Uncured Blends

**Figure 3:**
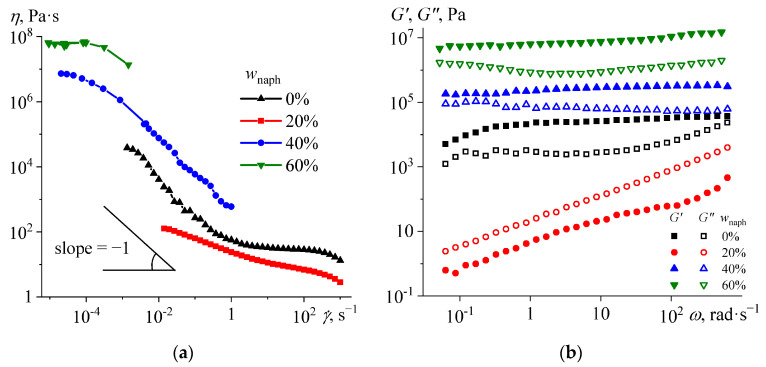
Dependences of viscosity on shear rate (**a**) and storage and loss moduli on angular frequency (**b**) for mixtures of epoxy resin, hardener, and naphthalene at 25 °C. The legends indicate the mass fraction of naphthalene.

The viscosity of complex heterogeneous mixtures may depend on the shear rate due to the deagglomeration of dispersed solids or destruction of their network structure [67,68]. In our case, naphthalene and the hardener are solid particles insoluble in epoxy resin at low temperatures. The uncured naphthalene-free composition has a shear-thinning behavior at 25 °C (Figure 3a), probably due to the breaking of agglomerates from hardener particles when the shear rate increases. Moreover, the dependence of viscosity has a slope of about −1 in log–log coordinates at low-to-moderate shear rates of 3·10^−4^–0.3 s^−1^, indicating the presence of yield stress [69], i.e., a shear stress destroying the percolation network of solid particles [70]. At lower shear rates than in the indicated range, the sample deforms without destroying the structural network, whereas the higher shear rates cause its flow with a broken structure [71,72].

Naphthalene at 20% mass fraction reduces the viscosity of the blend, which loses its yield stress but retains non-Newtonian behavior (Figure 3a). According to the phase diagram (Figure 2), the solubility of naphthalene at 25 °C is about 19%. Thus, 20% naphthalene almost completely dissolves within the epoxy resin, reducing its viscosity and decreasing the concentration of hardener particles versus the continuous epoxy medium. As a result, the hardener particles cease to form a spatial network but continue to agglomerate according to a shear-thinning behavior associated with the breakdown of agglomerates when the shear rate increases.

The situation changes at 40% naphthalene: The yield stress reappears, and the viscosity increases significantly. In this case, only half of the naphthalene dissolves within the continuous medium, while the other half probably agglomerates together with the hardener particles to form a united percolation network [72]. An increase in the naphthalene content to 60% further raises the viscosity, and the mixture stops flow at moderate and high shear rates that squeeze it out of the measuring gap due to edge fracture [73,74,75].

Frequency dependences of the storage modulus (characterizing elasticity) and loss modulus (related to internal friction, i.e., viscosity) can provide additional information on the structural state of mixtures. The base naphthalene-free system exhibits solid-like behavior since its storage modulus exceeds the loss modulus over the entire frequency range (Figure 3b). This behavior is typical for gels [76,77] and confirms the formation of a structural network by filler particles. The addition of 20% naphthalene destroys the spatial network, leading to a liquid-like behavior of the mixture, as both moduli become strongly dependent on the angular frequency, and the loss modulus exceeds the storage modulus. An increase in the naphthalene mass fraction up to 40–60% makes the moduli higher and weakly dependent on the angular frequency. In this case, the greater the naphthalene content, the higher the moduli, indicating the participation of naphthalene particles in structuring the systems demonstrating gel-like behavior. Naphthalene may integrate into a structural network of hardener particles, thereby increasing the density of interparticle contacts, or it may form its spatial network.

Thus, a naphthalene content of up to 20% reduces the viscosity of the resin/hardener blend, facilitating its processing and shaping. However, 40–60% of naphthalene conversely increases viscosity and yield stress because of its limited solubility and crystalline state at 25 °C. This point is negative, elevating the energy cost for mixing the compositions, which, however, can be overcome by increasing the temperature and converting the naphthalene to a dissolved state. In this case, the rheological behavior of the blends upon heating with their concomitant thermal curing becomes critical.

### 3.3. Thermal Curing of Blends

**Figure 4:**
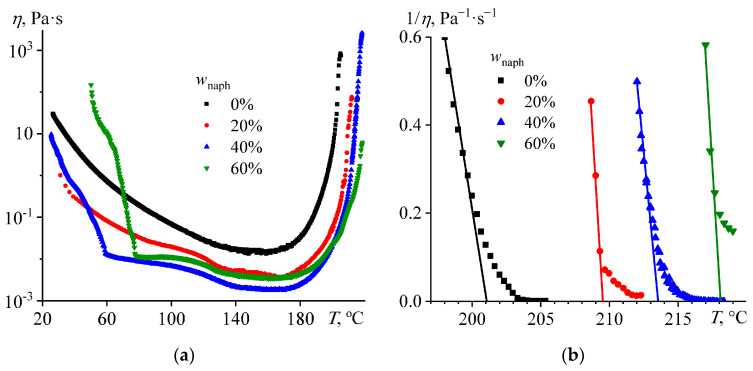
Viscosity during the curing of epoxy compositions in the mode of smooth temperature increase at a rate of 2 °C/min with a shear rate of 100 s^−1^ in normal (**a**) and reciprocal (**b**) coordinates. The legends indicate the mass fraction of naphthalene.

When the base naphthalene-free composition undergoes curing at a smooth temperature increase, its effective viscosity gradually decreases to a minimum at 145–160 °C (Figure 4a). At a further temperature increase, an intensive curing reaction starts, accompanied by a sharp growth in viscosity due to a rapid increase in the molecular weight of the forming epoxy polymer. Loss of fluidity and following sample detachment from the measuring surface occurs at 205 °C, when gelation occurs because of the appearance of a 3D network of chemical bonds. A more accurate position of the gel point can result from the linear extrapolation of the inverse viscosity (1/*η*) to zero, where the viscosity becomes infinitely large (Figure 4b). In the case of the base blend, the gelation temperature obtained by extrapolation is 201 °C (*T*_gel_, Table 1).

Naphthalene affects the effective viscosity of the compositions during the cross-linking process. A naphthalene content of 20–40% reduces viscosity over the entire range of curing temperatures because of the dissolution of less viscous naphthalene in the more viscous epoxy resin (0.9 mPa·s versus 52 mPa·s at 80 °C). Moreover, the viscosity of the 40% naphthalene-containing mixture is higher at low temperatures but conversely lower at high temperatures than for the 20% mixture. The explanation is that more than 19% of naphthalene dissolves within the epoxy medium only when heated, decreasing the viscosity. This effect is more noticeable at a naphthalene content of 60%, when the viscosity of the mixture at low temperatures exceeds the viscosity of all other mixtures and decreases sharply on heating due to the dissolution of naphthalene. The abrupt cessations of viscosity reduction occur at 59 °C and 78 °C for 40% and 60% naphthalene mass fractions, respectively, corresponding to the complete dissolution of naphthalene within the continuous epoxy medium. According to the phase diagram (Figure 2), 40% and 60% of naphthalene dissolve within the epoxy resin at 44 °C and 63 °C, respectively. The higher dissolution temperatures of naphthalene during the non-isothermal cross-linking may result from the slow dissolution of its crystals, in contrast to the phase diagram obtained under conditions close to equilibrium. After the total dissolution of naphthalene, the viscosity of the naphthalene-containing mixtures is 4–8 times lower than the viscosity of the base composition, and the mixture containing 40% naphthalene has the lowest viscosity. However, naphthalene slows the curing process slightly, maximally shifting the gelation point by 17 °C toward higher temperatures (*T*_gel_, Table 1). Gel-point displacement may result from the dissolution of naphthalene within the epoxy medium and the slowing of the curing reaction due to a decrease in the concentration of the reagents. The greater the naphthalene content, the higher the gelation temperature, as the concentrations of the epoxy resin and the hardener dissolved in the naphthalene decrease.

**Figure 5:**
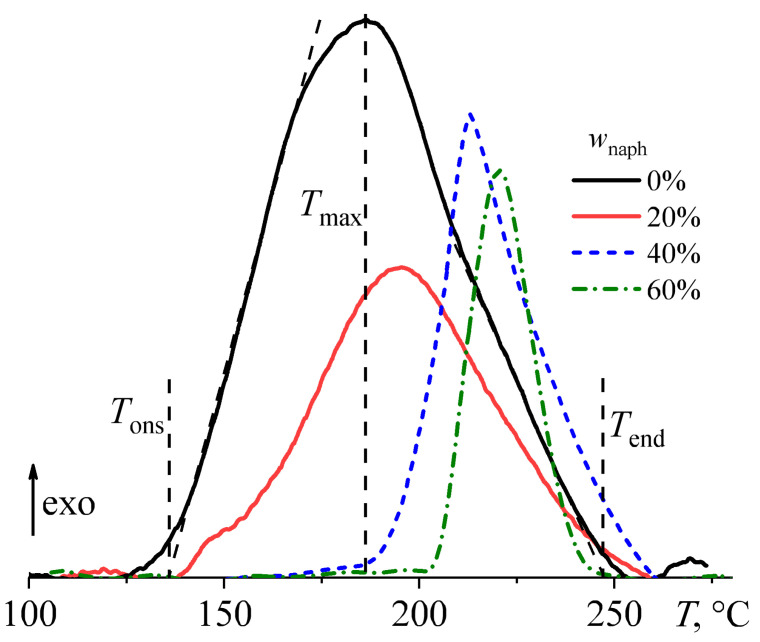
Heat flow during the curing of epoxy compositions at a heating rate of 2 °C/min. The legend indicates the mass fraction of naphthalene.

Calorimetry can provide additional information on the effect of naphthalene on epoxy curing (Figure 5). Analysis of heat flow change during the cross-linking process reveals a shift of heat-release maximum peaks toward high temperatures due to naphthalene (*T*_max_, Table 1). Naphthalene also significantly shifts the curing onset temperature (*T*_ons_) toward higher temperatures, confirming the retardation of cross-linking due to the presence of dissolved naphthalene. Meanwhile, naphthalene has little effect on the curing end temperature (*T*_end_). Thus, naphthalene gradually increases the onset and peak temperatures of the curing reaction and decreases its thermal effect, i.e., reduces the degree of completeness.

As the naphthalene concentration increases, the overall cross-linking thermal effect (Δ*H*, Table 1) is reduced due to the decreasing fraction of the epoxy resin and hardener in the mixture composition. The curing completeness can be evaluated using the reduced thermal effect of cross-linking [20,21]:

Δ*H*_red_ = 100·Δ*H*/(100 − *w*_naph_). (1)

Calculations of Δ*H*_red_ show that 20% naphthalene has no appreciable influence on curing completeness, whereas 40–60% decreases the reduced thermal effect of cross-linking by about one-third (300 J/g versus 203–215 J/g, Table 1). The decrease in the curing completeness may result from the absorption of hardener or epoxy resin by the arising naphthalene phase, which changes the resin/hardener ratio, making it non-stoichiometric.

Thus, naphthalene dissolves within the epoxy medium and decreases its cross-linking degree when added in high concentrations, as evidenced by the decline in the reduced thermal effect of the curing Δ*H*_red_. Both naphthalene dissolution and cross-linking reduction may significantly decrease the glass transition temperature of the cured polymer. In addition, it is unclear whether the naphthalene will remain dissolved after cross-linking or form a new phase because of a phase separation caused by the curing of the epoxy resin and its molecular weight growth. The effect of naphthalene on the phase transitions of the cured mixtures deserves detailed investigation.

### 3.4. Phase Transitions and Morphology of Cured Blends

**Figure 6:**
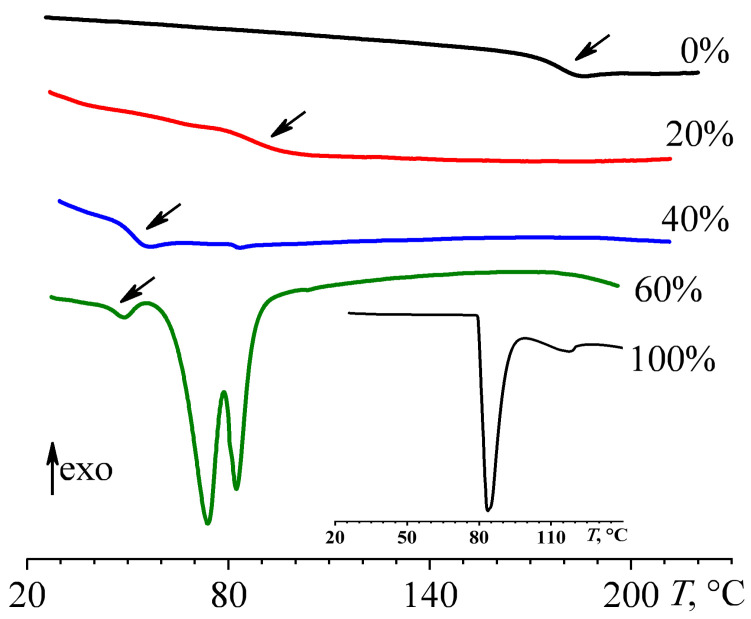
DSC thermograms of cured epoxy compositions containing naphthalene whose mass fraction is near the curves. The arrows indicate glass transitions. The inset shows data for pure naphthalene.

The thermogram of naphthalene-free cross-linked epoxy polymer displays the glass transition point at 178.2 °C (*T*_g_, Table 2), appearing as a step change in heat flow due to the change in heat capacity (Figure 6). Naphthalene at 20% mass fraction significantly reduces the glass transition temperature to 86.7 °C, indicating its dissolved state and plasticizing effect on the epoxy polymer. The absence of the melting point of naphthalene, which melts at 80–84 °C (inset to Figure 6), confirms its dissolved state. A naphthalene content as high as 40% plasticizes the epoxy polymer more, lowering its glass transition temperature to 51.4 °C. In this case, a weak endothermic transition (0.16 J/g) occurs at 83.2 °C due to the melting of dispersed naphthalene. An increase in the naphthalene content to 60% only slightly reduces the glass transition temperature to 46.4 °C but results in a distinct melting region of dispersed naphthalene in two steps at 73.8 °C and 82.3 °C (*T*_m_, Table 2). The higher temperature transition corresponds to the melting of pure naphthalene, whereas the lower one may be due to small sizes of naphthalene crystals or their contamination with epoxy resin, i.e., their defectiveness.

The nominal degree of naphthalene crystallinity in a cured blend can be estimated using the following equation:

(2) $$\mathrm{NDC}=\frac{\Delta H_{m}}{w_{\mathrm{naph}}\Delta H_{\mathrm{naph}}}\cdot 100\%$$

where Δ*H*_m_ is the melting enthalpy of the cured blend, whereas Δ*H*_naph_ is the melting enthalpy of pure naphthalene (151.6 J/g, see Table 2). A naphthalene content as low as 20% is entirely amorphous because of complete dissolution within the cured blend. At 40% naphthalene content, its nominal crystallinity degree is negligible (0.3%, Table 2), indicating its almost fully dissolved state. In turn, 60% of naphthalene has a nominal crystallinity degree of 46.8%, i.e., 28% of naphthalene in absolute value is crystalline, while 32% dissolves within the epoxy polymer. The decrease in the solubility of naphthalene from 40% to 32% when its content changes from 40% to 60% is due to a corresponding decline in the proportion of epoxy polymer dissolving naphthalene. In other words, 1 g of epoxy polymer dissolves about 0.7–0.8 g of naphthalene. Note that 1 g of epoxy resin dissolved about 0.23 g of naphthalene (i.e., 19%) at 25 °C before curing. These facts imply that the dissolved naphthalene in the cured epoxy polymer is metastable, as it is hard to expect an improvement in solubility during molecular weight growth and cross-linking of the epoxy resin. In other words, naphthalene forms a supersaturated solution resulting from high-temperature curing, and if not for the infinite viscosity of the cross-linked polymer, it would generate crystals. Nevertheless, despite unfavorable partial solubility, the maximum naphthalene content in the epoxy polymer (60%) and the efficiency in thermal energy storage (42.6 J/g) are much higher than for the same epoxy polymer but containing coconut oil (20%, 1.5 J/g), palm oil (20%, 6.4 J/g), or paraffin wax (45%, 20 J/g) as phase-change agents [20,21].

Thus, the maximum solubility of naphthalene in the cured epoxy polymer is about 40%. Its higher concentrations lead to phase separation of the curing mixture, which is homogeneous at high temperatures because of the complete miscibility of naphthalene and epoxy resin (see Figure 2). Phase separation occurs during the curing of epoxy resin, probably because of growth in its molecular weight, and leads to the release of naphthalene into a separate crystallizing dispersed phase.

**Figure 7:**
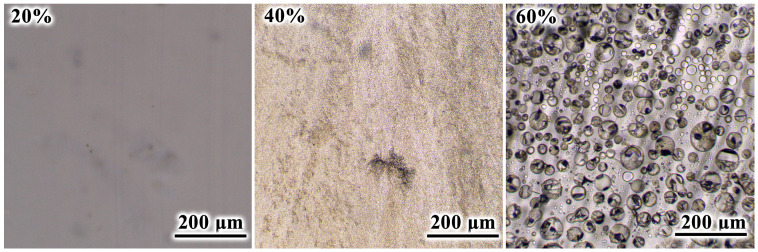
Microphotographs in a thin layer of cured epoxy compositions containing naphthalene whose mass fraction is indicated.

Because of the different phase states of the cured blends, their morphology changes significantly at various naphthalene contents (Figure 7). At 20% naphthalene content, the cured composition is optically homogeneous, implying complete dissolution of naphthalene. In the case of 40% naphthalene, there is fine dispersed heterogeneity at the optical visibility boundary, i.e., if naphthalene is present in the form of particles, they are of the order of 1 µm. At 60% of naphthalene, it appears as spherical droplets having diameters from 1–2 µm to 70 µm. A wide range in droplet size may indicate coalescence. In other words, little droplets form because of phase separation when naphthalene releases as a liquid phase at a high curing temperature of 180 °C. However, phase separation likely occurs before gelation, causing some smaller droplets to merge into larger ones.

**Figure 8:**
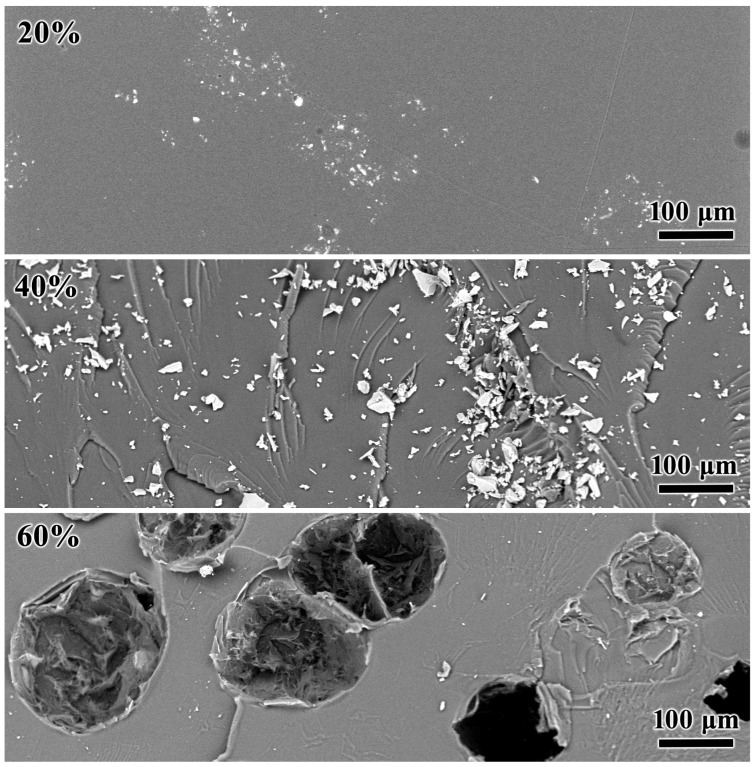
SEM images of the cross-sectional areas of cured epoxy compositions containing naphthalene whose mass fraction is indicated.

Scanning electron microscopy allows for examining the microstructure of the compositions in bulk and more detail (Figure 8). Even a mixture having as low as 20% naphthalene contains naphthalene microcrystals visible in cross-sectional chips as rare, small, white clusters with single-crystal sizes ranging from 0.5 to 6 µm. Likely, the cross-linked polymer as a supersaturated naphthalene solution releases these crystals at defect locations. At 40% naphthalene, its crystals grow to sizes from 0.5 to 20 µm and become more numerous. In turn, the blend containing 60% naphthalene has large spherical indentations, which probably remain from the detached naphthalene particles having sizes of 100–150 µm. However, optical microscopy showed that the droplet size in the same blend was only up to 70 μm (Figure 7, right). This inconsistency may be due to the curing for photographing in a thin (almost 2D) layer that does not allow the droplets to move and merge to larger sizes, like in the massive 3D sample for SEM examination. Meanwhile, the large size of naphthalene particles explains their better ability to store thermal energy compared to particles from vegetable oils or paraffin wax, which have smaller sizes (from 2 to 14 μm) and worse crystallizing because of the limited growth of crystals and their defectiveness [20,21,78]. The larger size of the naphthalene particles may result from a phase separation that occurred at a lesser epoxy-curing degree, a greater tendency of naphthalene droplets to merge, or a lower viscosity of the epoxy medium because of dissolved naphthalene.

Thus, naphthalene almost completely dissolves at its 20–40% content in cured epoxy blends, plasticizing them and lowering their glass transition temperature. Although these blends contain a small amount of naphthalene microcrystals, they do not reveal themselves from the practical viewpoint of thermal energy storage. At a mass fraction of 60%, about half of the naphthalene dissolves within the cured epoxy medium and plasticizes it. The other half manifests itself as large sphere-like particles able to crystallize and store thermal energy.

In summary, the epoxy resin allows for enclosing as much as 60% naphthalene, whose halves are in the dissolved metastable and coarse, dispersed states. From the viewpoint of fungicide material, the dissolved state of naphthalene is more desirable because it increases its evaporation rate from the epoxy polymer surface and allows its alteration by changing the initial naphthalene content. In contrast, dispersed naphthalene particles will work as a fungicide only after dropping the concentration of dissolved naphthalene within the cross-linked epoxy medium, which allows the crystalline naphthalene to dissolve, diffuse to the surface of the material, and then evaporate. In turn, dispersed naphthalene particles allow for the potential production of prolonged-acting fungicides, whose naphthalene crystals gradually replace pre-dissolved naphthalene during the use of the fungicide material. At the same time, from the viewpoint of phase change material, the maximum concentration of naphthalene and its crystalline state is desirable for better energy storage. In this respect, naphthalene works many times better in the epoxy medium than other organic phase-change agents (vegetable oils or paraffin wax) because of the higher maximum concentration (up to 60% vs. 20–45%) and fuller crystallization thanks to larger dispersed particles.

## 4. Conclusions

This first-time study of the rheological, thermophysical, and morphological features of epoxy blends containing 20–60% naphthalene before and after curing revealed the following:

- Naphthalene is fully soluble in uncured epoxy resin at 80 °C, whereas cooling causes its crystallization and drop in solubility to 19% at 25 °C;
- Before curing, 20% of naphthalene reduces the viscosity of the epoxy blend at 25 °C because of dissolution. However, its higher content conversely increases the viscosity and induces yield stress behavior and gelation because of the percolation network from naphthalene and hardener particles;
- Naphthalene reduces the viscosity during high-temperature curing of epoxy resin because of solubility, which delays the moment of gelation, lowering the rate and completeness of cross-linking because of the accompanying decrease in the concentrations of reactants diluted with naphthalene;
- About 40% of naphthalene metastably dissolves within the cured epoxy polymer, lowering its glass transition temperature, while its higher concentrations form coarse particles capable of crystallization and thermal energy storage.

Further work should aim at studying the evaporation rate of naphthalene from the epoxy polymer surface, determining the diffusion rate of naphthalene within the cross-linked epoxy polymer, and establishing effective concentrations of naphthalene that provide fungicidal action.

## Figures and Tables

**Table 1:** Temperatures of cure onset, heat release maximum, gelation, and curing end as well as thermal and reduced thermal cross-linking effects for epoxy compositions containing different mass fractions of naphthalene.

*w*_naph_, wt%	*T*_ons_, °C	*T*_max_, °C	*T*_gel_, °C	*T*_end_, °C	Δ*H*, J/g	Δ*H*_red_, J/g
0	136.0	186.3	201	247	300	300
20	152.4	195.2	210	248	243	303
40	193.8	213.0	214	254	129	215
60	211.1	217.0	218	237	81	203
Standard deviation	0.3	0.2	1	1	15	15

**Table 2:** The glass transition temperature of cured epoxy blends and melting temperature, melting enthalpy, and nominal crystallinity degree of their dispersed naphthalene.

*w*_naph_, wt%	*T*_g_, °C	*T*_m_, °C	Δ*H*_m_, J/g	NDC, %
0	178.2 ± 0.2	–	–	0
20	86.7 ± 0.2	–	–	0
40	51.4 ± 0.2	83.2 ± 0.2	0.16 ± 0.01	0.30 ± 0.01
60	46.4 ± 0.2	73.8 ± 0.2, 82.3 ± 0.2	42.6 ± 0.3	46.8 ± 0.2
100	–	83.5 ± 0.2 *	151.6 ± 0.7	100

* *T*_m_ is the position of the peak maximum; the onset of naphthalene melting occurs at 80 °C.

## Data Availability

The data presented in this study are available on request from the corresponding author.
